# ﻿The phylogeny and taxonomy of *Violella* (Tephromelataceae, lichenized Ascomycota), including a new species from China

**DOI:** 10.3897/mycokeys.121.155353

**Published:** 2025-09-02

**Authors:** Chun-jiao Zhong, Zun-tian Zhao, Ling Hu

**Affiliations:** 1 College of Geography and Environment, Shandong Normal University, Jinan, Shandong, 250014, China Shandong Normal University Jinan China; 2 Key Laboratory of Plant Stress Research, College of Life Sciences, Shandong Normal University, Jinan, Shandong, 250014, China Shandong Normal University Jinan China

**Keywords:** EF1-α gene, lichenized fungi, new combination, new taxa, Yunnan

## Abstract

*Violella yunnanensis* C. J. Zhong & L. Hu is described as new to science. It is characterized by its esorediate, areolate to weakly warted thallus, hymenium heavily pigmented with Fucatus-violet pigment, brownish inner ascospore walls, and its chemistry (atranorin and fumarprotocetraric acid). In addition, we collected specimens of *Mycoblastus sinensis* Kantvilas and *Violella wangii* T. Sprib. & Goffinet from the holotype localities. *Mycoblastus sinensis* is transferred to the genus *Violella* based on its morphology, chemistry and phylogeny, and it is proposed as *Violella sinensis* (Kantvilas) C. J. Zhong & L. Hu. The morphological descriptions, pictures and molecular phylogenetic analyses of the species are provided, along with a key to the *Violella* species known from world.

## ﻿Introduction

*Violella* T. Sprib. is a genus of lichenized fungi belonging to Tephromelataceae, Lecanorales, Lecanoromycetes, Ascomycota (Cannon et al. 2022; Wijayawardene et al. 2022). The family Tephromelataceae currently includes 4 genera (*Calvitimela*, *Mycoblastus*, *Tephromela* and *Violella*) and 53 species (Wijayawardene et al. 2022). The genus *Violella* was a group of mainly epiphytic species which was found in Western Europe, North America and the mountains of high Asia (Tønsberg 1992, 1993; James and Watson 2009; Spribille et al. 2010, 2011a, b). So far, *Violella* includes 2 species worldwide (Wijayawardene et al. 2022).

The genus *Violella* is separated from *Mycoblastus* (Spribille et al. 2011b) by an abundant Fucatus-violet pigment in the hymenium and inner ascospore walls that become brownish. In contrast, *Mycoblastus* typically has Cinereorufa-green pigments in the hymenium and hyaline ascospores. In addition, *Mycoblastus* has anastomosing paraphyses, making the hymenium coherent and fragmenting irregularly under pressure, even in K, while *Violella* has more columnar paraphyses with small bridges and tends to fragment along the columns (McCune 2007; Spribille et al. 2011b; Singh and Singh 2015; Cannon et al. 2022). In phylogenetic analysis, *Violella* was more closely related to *Calvitimela* and *Tephromela* than to *Mycoblastus* (Spribille et al. 2011b; Bendiksby et al. 2015; Fjelde et al. 2024). Prior to this study, only one species of *Violella* had been reported from China. *V. wangii* T. Sprib. & Goffinet was first described from Yunnan Province.

During a survey of the lichen diversity in the northwest of Yunnan Province in China, we collected numerous lichen specimens including *Violella wangii* and *Mycoblastus sinensis*, which were found in the type locality at Laojun Mountain and Yulong Mountain, respectively. During our research, a new species, *Violella yunnanensis* was discovered from Laojun Mountain and Meili Snow Mountain. Furthermore, we proposed a new combination *Violella sinensis* (Kantvilas) C. J. Zhong & L. Hu. In the present paper, we characterize these species using morphological, chemical, and molecular evidence and provide a key to the *Violella* species known from world.

## ﻿Materials and methods

### ﻿Morphological and chemical study

All the specimens were deposited in the Lichen Section of the Botanical Herbarium, Shandong Normal University, Jinan, China (**SDNU**). A dissecting microscope, Nikon SMZ 745T, was used to observe the morphological features. Apothecia and thalli were sectioned by hand with razor blade and their microscopic traits were observed and measured using an Olympus CX21 optical stereomicroscope. Photographs were taken by the Olympus SZX16 and BX16 microscope with DP72 camera system. Lugol’s iodine (I) was used to examine the apical structure of asci. Spots tests were conducted by K (a 10% aqueous solution of potassium hydroxide), C (a saturated aqueous solution of sodium hypochlorite), P (a saturated solution of p-phenylenediamine in 95% ethylalcohol). Secondary metabolites were detected by thin-layer chromatography (TLC) as described by Orange et al. (2001) in solvent C (toluene: acetic acid =170: 30). The dimensions of ascospores and the ascospore length/width ratio are presented as (minimum) mean±SD (maximum); n= the number of measurements.

### ﻿DNA extraction, amplification and sequencing

DNA was extracted from dry or fresh specimens. The voucher numbers refer to Table 1. The Sigma-Aldrich REDExtract-N-Amp Plant PCR Kit (St Louis, Missouri, USA) was used to isolate DNA, following the manufacturer’s instructions, except only 30 µl of extraction buffer and 30 µl dilution buffer were used. We selected two loci for this study: the nuclear ribosomal internal transcribed spacer region (nrITS) and one protein-coding gene, namely translation elongation factor 1-α (EF1-α). PCR amplification for nrITS and EF1-α was achieved using primers ITS1F and ITS4 (White et al. 1990), EF983 and EF2218R (Rehner and Buckley 2005), respectively. The 50 µl PCR mixture consisted of 2 µl DNA, 2µl of each primer, 25 µl 2× Taq PCR MasterMix (Taq DNA Polymerase [0.1 unit/µl]; 3 mM MgCl_2_; 100 mM KCl; 0.5 mM dNTPs; and 20mM Tris-HCl [pH 8.3]) (Tiangen, Beijing, China) and 19 µl ddH_2_O. Sequencing was performed by BioSune Biological Technology (Shanghai).

**Table 1.** Specimens and sequences used for phylogenetic analyses. Newly generated sequences are in bold.

Species	Locality	Voucher specimens	GenBank numbers	
			nrITS	EF1-α
*Calvitimela armeniaca* 1	Norway	O L-225775	OR763878	OR738421
*C. armeniaca* 2	Norway	O L-225781	OR763865	OR738420
*Tephromela atra* 1	Italy, Campania, Napoli, Capri Island	Muggia (TSB37119)	EU558648	JN009698
*T. atra* 2	Greece, Crete, Herakleion, Kameraki	Muggia (TSB37924)	EU558688	JN009697
*Violella fucata* 1	Germany, Bavaria, Bayerischer Wald	Spribille 32112 (GZU)	JN009732	–
*V. fucata* 2	USA, Mt. Greylock	Spribille 32161 (GZU)	JF744968	JN009703
*V. fucata* 3	Slovenia, Snežnik area	Spribille 30276 & Mayrhofer (GZU)	JN009733	–
***V. sinensis* 1**	**China, Mt. Yulong**	**SDNU20232737**	**PQ586228**	–
***V. sinensis* 2**	**China, Mt. Yulong**	**SDNU20232538**	**PQ586227**	**PQ602071**
***V. sinensis* 3**	**China, Mt. Meili Snow**	**SDNU20232001**	**PQ586226**	**PQ602070**
*V. wangii* 1	China, Mt. Laojun	Goffinet 10029 (KUN)	JN009734	JN009704
*V. wangii* 2	China, Mt. Laojun	Goffinet 10033 (UPS)	JN009735	–
***V. wangii* 3**	**China, Mt. Laojun**	**SDNU20232930**	**PQ586229**	**PQ602072**
***V. wangii* 4**	**China, Mt. Laojun**	**SDNU20232975**	**PQ586230**	**PQ602073**
***V. yunnanensis* 1**	**China, Mt. Meili Snow**	**SDNU20235026**	**PQ586223**	**PQ602076**
***V. yunnanensis* 2**	**China, Mt. Laojun**	**SDNU20232875**	**PQ586231**	**PQ602074**
***V. yunnanensis* 3**	**China, Mt. Laojun**	**SDNU20232882**	**PQ586232**	**PQ602075**

### ﻿Phylogenetic analysis

The raw sequences were initially checked with the BLAST tool on the NCBI online service (https://blast.ncbi.nlm.nih.gov/Blast.cgi) to ascertain that all the new sequences were reliable. All raw sequences were assembled and edited using Geneious 9.0.2. Sequences extracted from new materials were aligned with the additional sequence data from GenBank, using an online version of MAFFT v. 7.0.26. The algorithm of MAFFT chose Auto (FFT-NS-1, FFT-NS-2, FFT-NS-i or L-INS-i; depending on data size). We used *Tephromela atra* and *Calvitimela armeniaca* as the outgroup. Phylogenetic relationships were inferred using maximum likelihood (ML) and Bayesian inference (BI).

The best substitution models were estimated using ModerFinder v2.2.0 for the subsequent ML and BI analyses (Kalyaanamoorthy et al. 2017). In ML analysis, the best-fit model for nrITS and EF1-α were SYM+G and TRNEF+G, respectively. In BI analysis, GTR+I+G was the best-fit model for both sequences. The ML analyses were performed in IQ-TREE v2.2.0 with 1000 standard bootstrap replicates (Nguyen et al. 2014). The BI analyses were performed in MrBayes v.3.2.6 (Ronquist et al. 2012), using three Markov chains running for 2 million generations for the concatenated dataset. The trees were sampled every 100 generations, and the first 25% of the trees were discarded as burn-in. Bootstrap support (BS) ≥70 and posterior probability (PP) ≥0.95 were considered significant support values. The datasets/alignments were deposited in TreeBase (http://purl.org/phylo/treebase/phylows/study/TB2:S32226). All of the above analysis software were implemented in PhyloSuite v. 1.4.2 (Zhang et al. 2020; Xiang et al. 2023). The phylogenetic trees generated were visualized with FigTree v. 1.4.2 (Rambaut 2012).

## ﻿Results and discussion

**Figure 1.**
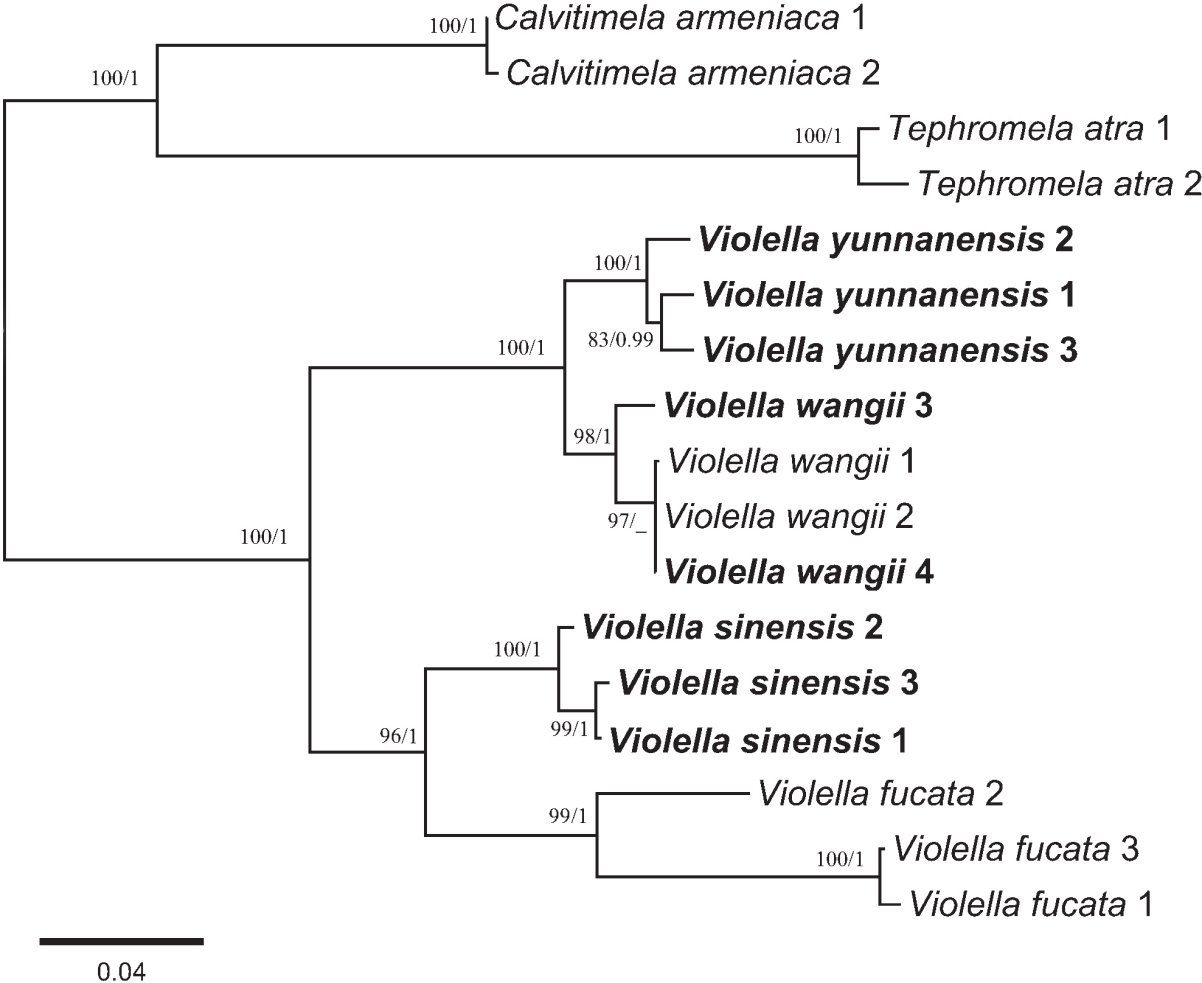
Phylogenetic tree generated from maximum likelihood (ML) analysis based on nrITS and EF1-α sequences. ML bootstrap values (left) and Bayesian posterior probabilities (right) are indicated at the nodes. The scale bar indicates the number of substitutions per site. Newly generated sequences from China are shown in bold.

In the present study we generated eight new nrITS and seven new EF1-α sequences (Table 1). We constructed ML and BI topologies based on these new sequences and fifteen additional sequences downloaded from GenBank, mostly from Spribille et al. (2011b) and Fjelde et al. (2024). The phylogenetic trees obtained from ML and BI exhibited consistent topologies, so only the ML tree was provided here (Fig. 1).

Within the phylogenetic tree, specimens of the putative new species formed a single clade with bootstrap support of 100 and a posterior probability of 1. This clade was strongly supported as sister to *V. wangii*. Based on the combination of morphological characters, chemistry and phylogenetic analysis, which are described in detail below (Table 2), we propose a new species named *Violella yunnanensis*. The new combination, *Violella sinensis*, is phylogenetically close to *V. fucata*, which is the type of the genus; they all contain atranorin and fumarprotocetraric acid as secondary metabolites, but *V. sinensis* is unequivocally non-sorediate and has well developed areolate, verruculose to papillose thallus, which distinguish it from *V. fucata*.

**Table 2.** Comparison of the characters of *Violella* species. (Spribille et al. 2011b; Kantvilas 2011; Cannon et al. 2022).

Species	Thallus	Apothecia (mm)	Ascospores (μm)	Chemistry
*V. fucata*	sorediate, soredia bluish grey	often absent	–	atranorin, fumarprotocetraric acid
	thin, effuse	rare, 0.5–1.5	(25–)30–48(–52) × 15–21	atranorin, fumarprotocetraric acid
*V. wangii*	sorediate, soredia white	often absent	–	atranorin, roccellic/angardianic acid
	granular and corticate areolate	(0.7–)1.3–2.6(–4.1)	(35–)41.7–54.2(–65) × (15–)20.8–30.8(–35)	atranorin, roccellic/angardianic acid
*V. sinensis*	esorediate, areolate, verruculose to papillose	0.5–1.25(–2.25)	(38–)45.95±6.18(–58) × (18–)25.65±4.63(–33)	atranorin, fumarprotocetraric acid
*V. yunnanensis*	esorediate, areolate to weakly warted	(0.4–)0.8–1.75(–2)	(43–)50.4±5.37(–63) × (23–)28.15±3.5(–38)	atranorin, fumarprotocetraric acid

### ﻿Taxonomy

#### ﻿ Violella sinensis

Taxon classificationFungiLecanoralesTephromelataceae

(Kantvilas) C. J. Zhong & L. Hu comb. nov.

0D45D31F-2151-5CD4-B5C9-4083221A4F46

Fungal Names: FN 572208

Fig. 2

≡ Mycoblastus sinensis Kantvilas, J. Jap. Bot. 86(2): 59 (2011).

##### Diagnosis.

The species is characterized by having esorediate, areolate, verruculose to papillose thallus, hymenium heavily pigmented with Fucatus-violet pigment, brownish inner ascospore walls, and its chemistry (atranorin and fumarprotocetraric acid).

##### Specimens.

China. Yunnan Prov.: • Yulong Co., Baisha Vil., entrance of Alpine Botanical Garden, 27°0'10.80"N, 100°10'49.50"E, 3204 m, on bark, 28 April 2023, L. Hu et al. SDNU 20232538.

##### Description.

Thallus crustose, chalk white, ochre to grey, areolate, consisting of discrete convex to bullate areoles (0.25–)0.37–0.5 mm diam., forming a verruculose to papillose, widespread crust. Medulla white, with crystals. Soredia and isidia absent. Prothallus visible, dark. Photobiont chlorococcoid, cells rounded to irregularly angular, 8–16 μm diam.

Apothecia rounded, single or clustered in groups of 2–3 and becoming confluent, 0.5–1.25(–2.25) mm diam., base broadly adnate; disc ± flat to strongly convex, black and shiny, becoming cracked when old; margin indistinct. Proper exciple reduced, similar in structure to the hymenium, hyphae radiate, similar to paraphyses. Epihymenium not differentiated as a distinct layer. Hymenium 80–150 μm tall, densely inspersed with minute oil droplets, strongly infused with Fucatus-violet pigment, especially in the upper part, sometimes also infused with Cinereorufa-green, K+ peacock green. Subhymenium consisting of a thin layer of ascogenous hyphae, 17.5–27.5 μm tall, filled like the hymenium with Fucatus-violet pigment but sometimes also infused with Cinereorufa-green. “thalline cushion” in section prosoplectenchymatous, variable in thickness, 12.5–137.5 μm thick. Hypothecium absent. The amyloid reaction is restricted to the asci and surrounding gel, and does not occur through the whole hymenium. Paraphyses branched and anastomosing, 2–4 μm wide, arranged vertically and linked to each other in their lower halves by thin bridges; paraphyses tips not or scarcely expanded. Asci clavate, *Biatora*-type. Ascospores ovate to broadly ellipsoid, 2 per ascus, beginning colourless and apparently with a single wall, eventually developing a secondary inner wall, which quickly turns brown while still in ascus; outer wall thick, to 4 μm in some cases, the inner brown wall thin, healthy ascospores (38–)45.95±6.18(–58) × (18–)25.65±4.63(–33) μm in water, length/ width ratio (1.37–)1.83±0.29(–2.28); n=20. Pycnidia not seen in our materials.

##### Chemistry.

Spot test: thallus K+ yellow, C–, P+ orange red, UV–. TLC: atranorin and fumarprotocetraric acid.

##### Ecology and distribution.

Found on twigs of *Rhododendron* and bark in subalpine and alpine regions. Collections came from elevations of 3200–3800 m in the northwest of Yunnan Province in China. Some collections were associated with *Mycoblastus affinis* and *Ochrolechia*.

##### Notes.

The species is characterized by having esorediate, areolate, verruculose to papillose thallus, hymenium heavily pigmented with Fucatus-violet pigment, brownish inner ascospore walls, and its chemistry (atranorin and fumarprotocetraric acid). It is distinct from *V. wangii* in its chemistry (fumarprotocetraric acid instead of roccellic/angardianic acid) and thallus morphology (esorediate and verruculose to papillose, instead of sorediate and composed of granular, corticate areoles) (Spribille et al. 2011b). *V. sinensis* was also different with the chemically concordant *V. fucata*. Apothecia were always present in the former, while rarely found in the later; thallus was thick, esorediate, areolate and verruculose to papillose in the former, while thin, sorediate, effuse in the later (Spribille et al. 2011b).

*Mycoblastus sinensis* was originally collected in Yunnan Province at Yulong Mountain. It was placed in the genus *Mycoblastus* based on its relatively large apothecia and *Biatora*-type asci with two, thick-walled ascospores (Kantvilas 2011). After the collection of fresh specimens from the same locality and comparison with the photograph and description of the type specimen, we confirmed that our collections were morphologically identical to the holotype, except that *M. sinensis* had larger (0.7–2.5(–3.5) mm) white-rime apothecia (Kantvilas 2011). However, phylogenetic analyses demonstrated that this species should be placed within the genus *Violella* rather *Mycoblastus*. In addition, this species is more closely matches the description of *Violella* (Kantvilas 2011). We consider that the type specimens were previously misassigned as species of *Mycoblastus*. It is likely that Kantvilas didn’t recognize the establishment of this new genus *Violella* because it was published in the same year as the other one (Spribille et al. 2011b; Kantvilas 2011). Based on the morphology, chemistry, and phylogeny of specimens from the type locality, a new combination is proposed here, *Violella sinensis*.

**Figure 2.**
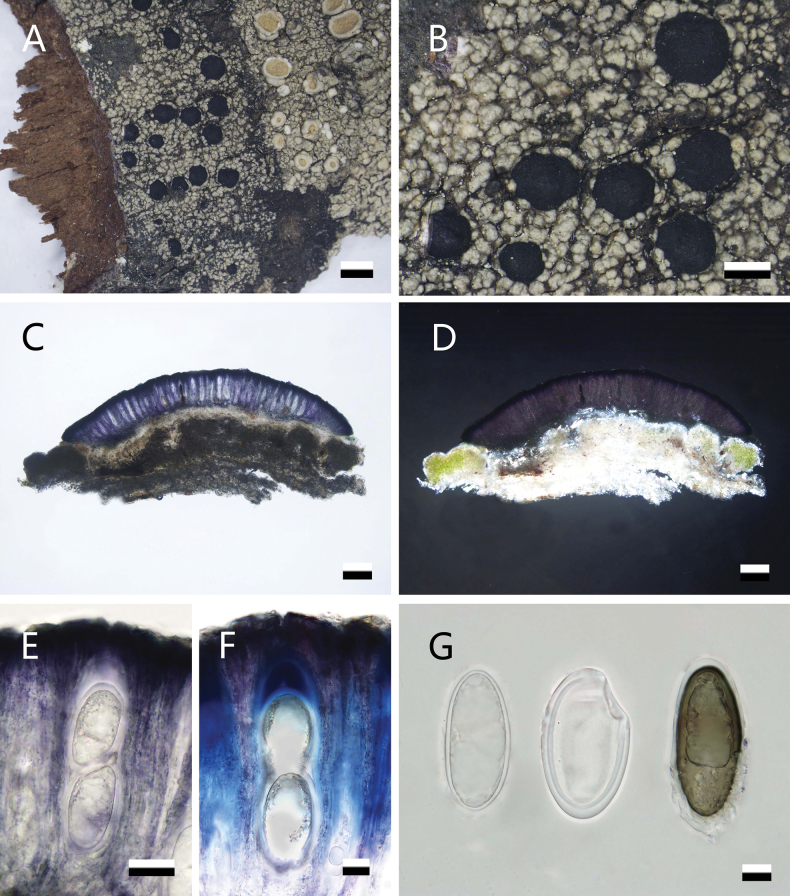
*Violella sinensis* (SDNU 20232538). **A.** Thallus; **B.** Apothecia; **C.** Apothecium section; **D.** Crystals; **E.** Ascus; **F.** Amyloid reaction of ascus; **G.** Ascospores. Scale bars: 1 mm (**A**); 500 μm (**B**); 100 μm (**C, D**); 20 μm (**E**); 10 μm (**F, G**).

##### Specimens examined.

China. Yunnan Prov.: • Diqing Tibetan Autonomous Prefecture, Deqin Co., on the way from Yubeng Upper Village to Glacier Lake beside the observation platform in Meili Snow Mountain National Park, 28°23'56.37"N, 98°46'8.97"E, 3506 m, on bark, 22 April 2023, L. Hu, C.J. Zhong & J.X. Wang. SDNU 20232001, 20235022, 20235025. • Lijiang, Yulong Co., on the mountain next to Yulong Lake in Gaoshan Botanical Garden, 27°0'12.89"N, 100°11'8.85"E, 3228 m, on bark, 27 April 2023, L. Hu, C.J. Zhong & J.X. Wang. SDNU 20232503, 20235064, 20235065. • Baisha Vi., entrance of Alpine Botanical Garden, 27°0'10.80"N, 100°10'49.50"E, 3204 m, on bark, 28 April 2023, L. Hu, C.J. Zhong & J.X. Wang. SDNU 20232538, 20232542. • Alpine Botanical Garden, 27°0'25.17"N, 100°10'34.51"E, 3399 m, on bark, 28 April 2023, L. Hu, C.J. Zhong & J.X. Wang. SDNU 20232559. • hidden slope of Alpine Botanical Garden, 27°0'23.91"N, 100°10'41.05"E, 3421 m, on bark, 29 April 2023, L. Hu, C.J. Zhong & J.X. Wang. SDNU 20235063, 20232737, • same location, 27°0'30.65"N, 100°10'29.75"E, 3475 m, on bark, 29 April 2023, L. Hu, C.J. Zhong & J.X. Wang. SDNU 20232753. • same location, 27°0'39.77"N, 100°10'39.12"E, 3511 m, on bark, 29 April 2023, L. Hu, C.J. Zhong & J.X. Wang. SDNU 20232767. • Yulong Co., next to Laojun Mountain Homestay, 26°37'54.05"N, 99°43'37.23"E, 3821 m, on bark, 30 April 2023, L. Hu, C.J. Zhong & J.X. Wang. SDNU 20232873.

#### ﻿ Violella yunnanensis

Taxon classificationFungiLecanoralesTephromelataceae

C. J. Zhong & L. Hu sp. nov.

9222E499-BFF7-5C2B-B637-9334EA5A2FB0

Fungal Names: FN 572207

Fig. 3

##### Diagnosis.

The species is characterized by having esorediate, areolate to weakly warted thallus, hymenium heavily pigmented with Fucatus-violet pigment, brownish inner ascospore walls, and its chemistry (atranorin and fumarprotocetraric acid).

##### Type.

China. Yunnan Prov.: • Deqin Co., on the way from Yubeng Upper Village to Glacier Lake beside the observation platform in Meili Snow Mountain National Park, 28°23'56.37"N, 98°46'8.97"E, 3506 m, on bark, 22 April 2023, L. Hu et al. SDNU 20235026 (holotype).

##### Description.

Thallus crustose, areolate, weakly warted, consisting of discrete flattened to convex areoles (0.18–)0.23–0.28 mm diam., color grey to pale ocher, with crystals in the medulla. Soredia and isidia absent. Hypothallus not observed. Photobiont chlorococcoid, cells rounded to irregularly angular, (7–)8–10(–12) μm diam.

Apothecia rounded to irregular, single or clustered in groups of 2–3 and becoming confluent, (0.4–)0.8–1.75(–2) mm diam., base broadly adnate; disc ± flat to weakly convex, jet black and shiny; margin indistinct; “thalline cushion” present, visible from above and forming a thin white line at least when young, in section prosoplectenchymatous, variable in thickness, 25–100(–137.5) μm thick, clearly differentiated from subhymenium above and medulla below. Proper exciple similar in structure to the hymenium, hyphae radiate, similar to paraphyses, filled with Fucatus-violet pigment and often suffused with Cinereorufa-green. Epihymenium not differentiated. Hymenium 125–137.5 μm tall, densely inspersed with minute oil droplets; the Fucatus-violet pigment is concentrated at the base and the top of the hymenium and largely diffuse. Subhymenium consisting of a thin layer of ascogenous hyphae, 30–32.5 μm tall, filled like the hymenium with Fucatus-violet pigment but sometimes also infused with Cinereorufa-green pigment; differentiated hypothecium absent. Paraphyses branched, anastomosing, 2–2.5 μm wide; paraphyses’ tips not or scarcely expanded, 2.5–3 μm wide. Asci clavate, inner and outer walls staining blue, tholus strongly I+ blue, pierced by a broad, conical non-amyloid structure, thus similar to the *Biatora*-type. Ascospores ellipsoidal, 2 per ascus (occasionally 1), beginning colourless and apparently with a single wall, eventually developing a secondary inner wall, which quickly turns brown while still in the ascus; outer wall thick, to 3 μm in some cases, the inner brown wall thin, often collapsing, live, healthy ascospores also with brown endospore, (43–)50.4±5.37(–63) × (23–)28.15±3.5(–38) μm in water, length/ width ratio (1.53–)1.8±0.14(–2); n=20. Pycnidia not seen in Chinese materials.

**Figure 3.**
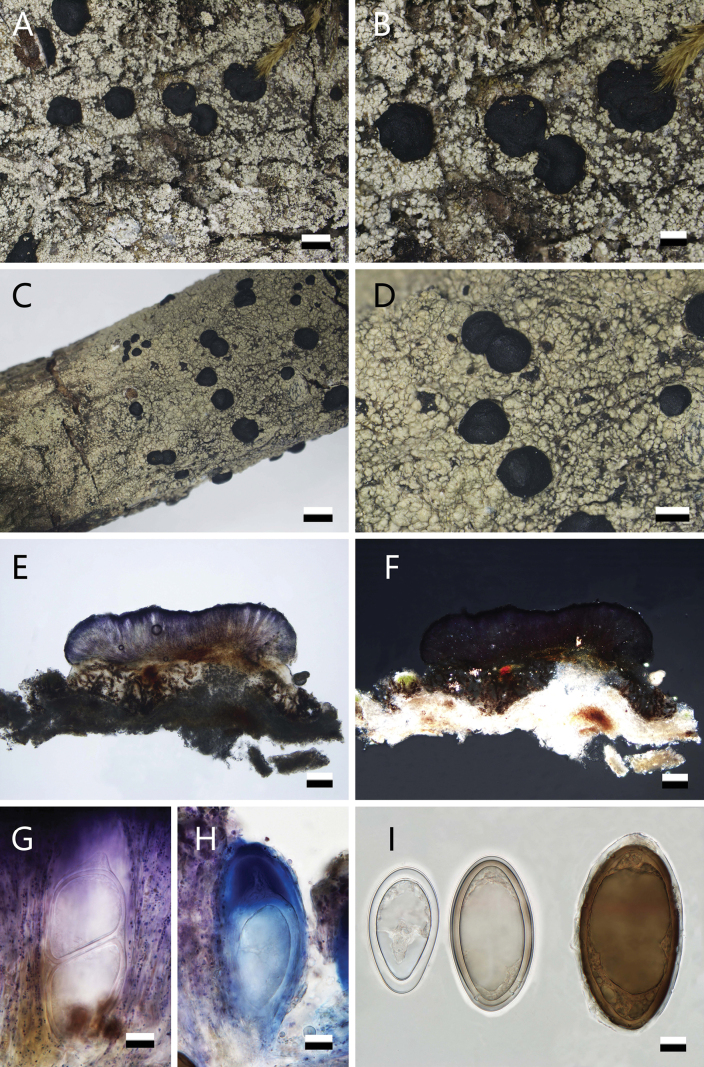
*Violella wangii* (**A, B.**SDNU 20232975). **A.** Thallus; **B.** Apothecia. *Violella yunnanensis* (**C–I.** Holotype! SDNU 20235026). **C.** Thallus; **D.** Apothecia; **E.** Apothecium section; **F.** Crystals; **G.** Ascus; **H.** Amyloid reaction of ascus; **I.** Ascospores. Scale bars: 1 mm (**A, C**); 500 μm (**B, D**); 100 μm (**E, F**); 10 μm (**G–I**).

##### Chemistry.

Spot test: thallus K+ yellow, C–, P+ orange-red, UV–; TLC: atranorin and fumarprotocetraric acid.

##### Ecology and distribution.

Found on twigs of *Rhododendron* and bark in subalpine and alpine regions. Collections came from elevations of 3500–3800 m in the northwest of Yunnan Province in China.

##### Etymology.

The specific epithet ‘yunnanensis’ refers to the Yunnan Province, where this species was found.

##### Notes.

*Violella yunnanensis* is characterized by having esorediate, areolate to weakly warted thallus, hymenium heavily pigmented with Fucatus-violet pigment, brownish inner ascospore walls, and producing atranorin and fumarprotocetraric acid. *Violella wangii* has a similar habitat to this new species. Specimens of *V. wangii* collected at Laojun Mountain are found growing on bark of *Rhododendron* sp. or on wood of *Pinus* at elevations ranging from 3400–3900 m. However, *Violella wangii* differs in its chemistry (atranorin and roccellic/angardianic acid), thallus morphology (white, granular corticate areoles, sorediate) and larger apothecia [(0.7–)1.3–2.6(–4.1) mm] (Fig. 3A, B). *V. fucata* differs from *V. yunnanensis* in possessing a generally thin, effuse, sorediate thallus, apothecia rare and smaller ascospores [(38.5±6.7×18.5±3.3μm, n=24) vs (43–)50.4±5.37(–63) × (23–)28.15±3.5(–38) μm, n=20)] (Spribille et al. 2011b). Furthermore, *Violella yunnanensis* differs from *Violella sinensis* in its weakly warted thallus and larger ascospores.

##### Specimens examined.

China. Yunnan Prov.: • Diqing Tibetan Autonomous Prefecture, Deqin Co., on the way from Yubeng Upper Village to Glacier Lake beside the observation platform in Meili Snow Mountain National Park, 28°23'56.37"N, 98°46'8.97"E, 3506 m, on bark, 22 April 2023, L. Hu, C.J. Zhong & J.X. Wang. SDNU 20231997, 20232019, 20235026. • Lijiang, Yulong Co., Baisha Vi., Alpine Botanical Garden, 27°1'2.33"N, 100°10'27.11"E, 3708 m, on bark, 28 April 2023, L. Hu, C.J. Zhong & J.X. Wang. SDNU 20232618. • Yulong Co., next to Laojun Mountain Homestay Vi., 26°37'54.05"N, 99°43'37.23"E, 3821 m, on bark, 30 April 2023, L. Hu, C.J. Zhong & J.X. Wang. SDNU 20232874, 20232841, 20232885, 20232828, 20232875, 20232890, 20232882.

### ﻿Key to the species of *Violella* in the world

**Table:** 

1	Soralia present; apothecia often absent	**2**
1	Soralia absent; apothecia present	**3**
2	Soredia often bluish grey; thallus Pd+orange-red	***V. fucata***
2	Soredia white; thallus Pd–	***V. wangii***
3	Thallus K+yellow, Pd–	***V. wangii***
3	Thallus K+yellow, Pd+orange-red	**4**
4	Thallus thin, effuse; ascospores (25–)30–48(–52) × 15–21 μm	***V. fucata***
4	Thallus well developed; ascospores larger	**5**
5	Areolate, verruculose to papillose thallus; ascospores (38–)45.95±6.18(–58) × (18–)25.65±4.63(–33) μm	***V. sinensis***
5	Areolate to weakly warted thallus; ascospores (43–)50.4±5.37(–63) × (23–)28.15±3.5(–38) μm	***V. yunnanensis***

## Supplementary Material

XML Treatment for Violella sinensis

XML Treatment for Violella yunnanensis

## ﻿Additional information

### Use of AI

No use of AI was reported.

### Author ORCIDs

Chun-jiao Zhong https://orcid.org/0009-0001-5159-238X

Ling Hu https://orcid.org/0009-0008-4568-6639

### Data availability

All of the data that support the findings of this study are available in the main text.
